# Momentary Assessment of Adults’ Physical Activity and Sedentary Behavior: Feasibility and Validity

**DOI:** 10.3389/fpsyg.2012.00260

**Published:** 2012-07-30

**Authors:** Genevieve Fridlund Dunton, Yue Liao, Keito Kawabata, Stephen Intille

**Affiliations:** ^1^Department of Preventive Medicine, University of Southern CaliforniaLos Angeles, CA, USA; ^2^College of Computer and Information Science, Northeastern UniversityBoston, MA, USA; ^3^Bouvé College of Health Sciences, Northeastern UniversityBoston, MA, USA

**Keywords:** physical activity, sedentary behavior, ecological momentary assessment, validity, adults, accelerometers

## Abstract

**Introduction:** Mobile phones are ubiquitous and easy to use, and thus have the capacity to collect real-time data from large numbers of people. Research tested the feasibility and validity of an Ecological Momentary Assessment (EMA) self-report protocol using electronic surveys on mobile phones to assess adults’ physical activity and sedentary behaviors. **Methods:** Adults (*N* = 110; 73% female, 30% Hispanic, 62% overweight/obese) completed a 4-day signal-contingent EMA protocol (Saturday–Tuesday) with eight surveys randomly spaced throughout each day. EMA items assessed current activity (e.g., Watching TV/Movies, Reading/Computer, Physical Activity/Exercise). EMA responses were time-matched to minutes of moderate-to-vigorous physical activity (MVPA) and sedentary activity (SA) measured by accelerometer immediately before and after each EMA prompt. **Results:** Unanswered EMA prompts had greater MVPA (±15 min) than answered EMA prompts (*p* = 0.029) for under/normal weight participants, indicating that activity level might influence the likelihood of responding. The 15-min. intervals before versus after the EMA-reported physical activity (*n* = 296 occasions) did not differ in MVPA (*p* > 0.05), suggesting that prompting did not disrupt physical activity. SA decreased after EMA-reported sedentary behavior (*n* = 904 occasions; *p* < 0.05) for overweight and obese participants. As compared with other activities, EMA-reported physical activity and sedentary behavior had significantly greater MVPA and SA, respectively, in the ±15 min of the EMA prompt (*p*s < 0.001), providing evidence for criterion validity. **Conclusion:** Findings generally support the acceptability and validity of a 4-day signal-contingent EMA protocol using mobile phones to measure physical activity and sedentary behavior in adults. However, some MVPA may be missed among underweight and normal weight individuals.

## Introduction

Participating in regular physical activity has been shown to significantly reduce the risk of cancer, diabetes, and heart disease (Physical Activity Guidelines Advisory Committee, 2008). However, recent estimates suggest that only 10% of U.S. adults meet the Physical Activity Guidelines for Americans of at least 150 min of moderate-to-vigorous intensity physical activity per week (Tucker et al., 2011). Also, rates of physical activity decline across adulthood for men and women regardless of racial/ethnic group (Hawkins et al., 2009). Studies examining physical activity determinants, health consequences, and promotion strategies rely upon accurate and unbiased physical activity measures (Haskell, 2012; Troiano et al., 2012). Recall-based self-report methods of assessing physical activity can be prone to errors and biases (Merom et al., 2009; Loney et al., 2011; Nicaise et al., 2011). Objective physical activity measures such as accelerometers and pedometers are unable to provide information about specific activity types (e.g., watching TV versus computer use), suffer from substantial missing data due to non-wear (Troiano et al., 2008; Colley et al., 2010) and are unable to measure mood during or the context of physical activity, which may be important factors that influence behavior. Technology-enabled real-time self-report assessment strategies can overcome many of these limitations.

Recent advances in mobile phone technology have created opportunities for Ecological Momentary Assessment (EMA) of physical activity and sedentary behaviors in naturalistic settings (Patrick et al., 2008; Dunton and Atienza, 2009). Mobile phones have become affordable, easy to use, and quite ubiquitous. An estimated 68% of adults worldwide own a mobile phone^1^, and they have been widely adopted across socioeconomic groups and in developing countries (Kaplan, 2006; Kosaraju et al., 2010). Software applications can be loaded onto basic mobile phones or smartphones to trigger electronic EMA surveys in real time. Some preliminary evidence for the utility of EMA to measure physical activity is available (Rofey et al., 2010; Dunton et al., 2011; Thomas et al., 2011). EMA can be used to measure physical activity and sedentary behaviors alone or in combination with accelerometers, Global Positioning Systems (GPS), and heart-rate and respiration monitors. An added benefit of EMA is the capability to simultaneously measure contextual, social, or psychological factors that may influence physical activity such as environmental perceptions, social companionship, mood, and stress (e.g., Dunton et al., 2009; Kanning and Schlicht, 2010; Conroy et al., 2011).

The goals of this study were to test the feasibility, acceptability, and validity of a real-time EMA protocol using self-report electronic surveys on mobile phones to measure adults’ physical activity and sedentary behaviors in naturalistic settings. The first objective sought to describe the rates of mobile phone damage and loss, technical problems, and EMA survey response. The second objective was to determine whether participants engaged in higher levels of moderate-to-vigorous physical activity (MVPA) during unanswered EMA survey prompts, which would indicate that participants either do not carry the mobile phone or do not answer the EMA survey prompts during physical activity. The third objective was to determine whether the act of completing the EMA survey interrupted physical activity or sedentary behavior (i.e., activity levels differed before and after completing the survey). The fourth objective was to evaluate the criterion validity of the EMA self-reports of physical activity and sedentary behavior by comparing with time-matched data collected through an accelerometer. All objectives were initially stratified by the weight status because there is some evidence to suggest that overweight and obese individuals perform different types of physical activity than normal weight individuals (Spees et al., 2012). The overall purpose of the EMA protocol was not to provide a measure of total physical activity and sedentary behavior during the monitoring period. Instead, the goal was to sample the occurrence of specific behaviors that can be linked to other time-intensive EMA measures such as mood and context.

## Materials and Methods

### Participants and recruitment

Participants included healthy adults living in and around Chino, California (about 30 miles east of downtown Los Angeles). The current study analyzed baseline data from a longitudinal study called Project Measuring Our Behaviors in Living Environments (MOBILE), which is investigating the effects of environmental and intrapersonal factors on health behavior decision-making processes. Recruitment occurred through a number of channels including posters placed at community locations, letters sent to places of residence, and references from other research studies. Inclusion criteria consisted of the following: (a) age of 28 years or older, (b) living in Chino, CA, or a surrounding community, and (c) able to answer electronic EMA surveys while at work. Participants were excluded who (a) did not speak and read fluently in English (b) had an annual household income greater than $210,000, (c) regularly performed less than 150 min per week of exercise or physical activity, and (d) had physical limitations making them unable to exercise. Individuals who met the eligibility criteria were scheduled for a data collection appointment at a local community site or their home. This research was reviewed and approved by the Institutional Review Board at the University of Southern California.

### Ecological momentary assessment

**Figure 1:**
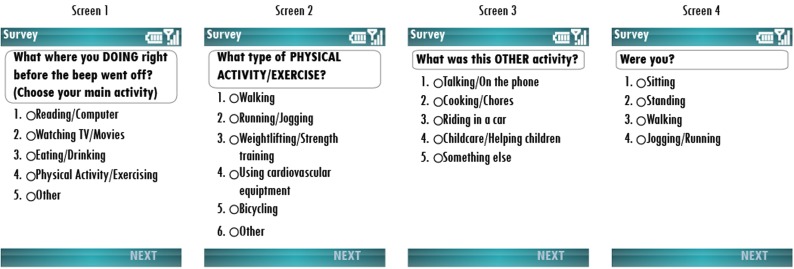
**Ecological momentary assessment (EMA) screen shots**. Images display how Ecological Momentary Assessment (EMA) items and response choices appeared on the display screen of the mobile phone. Respondents used the key pad to toggle up/down and select their response. Only one response choice could be selected per screen. If a respondent selected “Physical Activity/Exercising” on Screen 1, he/she was automatically directed to Screen 2. If a respondent selected “Other” on Screen 1, he/she was automatically directed to Screen 3. If a respondent selected “Something else” on Screen 3, he/she was automatically directed to Screen 4.

This study used electronic EMA to measure participants’ current type of activity at any given moment. Other EMA items assessed social and physical context, mood and stress, but those variables were not the focus of the current paper. Data were collected using an HTC Shadow mobile phone (T-Mobile USA, Inc.) with a custom version of the MyExperience software installed^2^ (Froehlich et al., 2007). The mobile phone calling, texting, and internet capabilities were disabled. The software was programmed to display electronic question sequences and response choices on mobile phone screen (see Figure 1).

Participants used the up and down arrows on the phone key pad to select a response choice from the options provided. Data were stored on the phone in an electronic file until downloaded by researchers. Verbal and written instructions were provided on how to use the device. Prior to starting the study, participants completed a practice assessment in the presence of a research staff member and were given the opportunity to ask questions.

**Figure 2:**
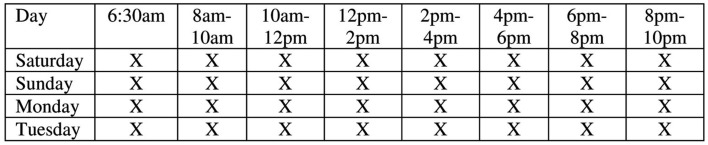
**Ecological momentary assessment (EMA) procedure**. Each X represents an EMA survey that was prompted at a random time with the specified time interval.

Participants were monitored across 4 days (Saturday–Tuesday) between 6:30 am and 10:00 pm. Eight EMA surveys were prompted per day. Each EMA survey was prompted at a random time within eight pre-programmed windows in order to ensure adequate spacing across the day (see Figure 2). Participants were asked to carry the mobile phone while at work and answer surveys when prompted. EMA surveys were prompted using an auditory signal. Phones could be set to vibrate mode in order to avoid disrupting activities. Upon receiving a phone signal, participants were instructed to stop their current activity and complete a short electronic EMA question sequence. This process required 2–3 min. If a signal occurred during an incompatible activity (e.g., sleeping or bathing), participants were instructed to ignore it. If no entry was made, the phone emitted up to three reminder signals at 5 min intervals. After this point, the electronic EMA survey became inaccessible until the next recording opportunity. During the monitoring period, participants received one phone call and one to two SMS messages from researchers to inquire about any technical problems and remind them to recharge the phone each night. A study hotline was also available to participants to report technical issues and request a replacement phone if necessary. Participants were compensated up to $50 for participating in the study: $18 plus an additional $1 for each completed EMA survey entry (32 total) over the 4-days.

### Measures

#### EMA items

Each EMA question sequence measured participants’ current activity type [“What were you DOING right before the beep went off (Choose your main activity)?”; see Figure 1]. Two automatic branching question sequences were programmed according to the response of this initial question. For the first branching sequence, if “Physical Activity/Exercise” was selected in response to the original question, the participant received the follow-up question, “what type of PHYSICAL ACTIVITY/EXERCISE?” In the second branching sequence, if a participant responded “Other,” he/she received the follow-up question, “what was this OTHER activity?” Subsequently, if he/she indicated, “Something else,” the question “were you (Sitting, Standing, Walking, Jogging/Running)?” was shown. For data analyses testing the first through third study objectives, responses indicating “Physical Activity/Exercise” and “Jogging/Running” were coded as *physical activity* and those indicating “Reading/Computer,” “Watching TV/Movies,” and “Sitting” were coded as *sedentary behavior*. All activities were examined separately for analyses testing the fourth study objective. The EMA items were administered in English.

#### Physical activity

The Actigraph, Inc., GT2M model activity monitor (firmware v06.02.00) provided an objective measure of physical activity. The device was worn on the right hip attached to an adjustable belt. Participants were asked to wear the accelerometers across seven continuous days (encompassing the 4-days of EMA monitoring). The devices were not worn when sleeping, bathing, or swimming. The cut-point for MVPA was consistent with studies of national surveillance data (Troiano et al., 2008; Belcher et al., 2010). The MVPA threshold was 2020 counts per minute (equivalent to three METs). Sedentary activity (SA) was defined as less than 100 counts per minute (Healy et al., 2008). All accelerometer recordings were time-stamped in order to be linked with EMA data captured on the mobile phone. The internal clocks of the mobile phone and accelerometer were both synchronized to the same computer. A time window was created around each EMA survey, which comprised the 15-min before and the 15-min after the survey prompt. EMA entries with a total of zero activity counts in the 15-min before and 15 min after the survey prompt were considered accelerometer non-wear and excluded from analyses.

#### Height and weight

Height and weight were measured in duplicate using an electronically calibrated digital scale (Tanita WB-110A) and professional stadiometer (PE-AIM-101) to the nearest 0.1 kg and 0.1 cm, respectively. Body Mass index (BMI) was calculated (kg/m^2^). Weight status was classified as follows: under and normal weight (BMI < 25), overweight (BMI greater than or equal to 25 and less than 30), and obese (BMI ≥ 30).

#### Demographic and time variables

Participants’ age, sex, ethnicity, and annual household income were assessed through a self-report paper-and-pencil survey. Annual household income was coded into quartiles (less than $40,000, $40,000–$70,000, $70,001–$90,000, above $90,000). Each EMA survey was also coded for the time of day that it occurred [i.e., morning (6:30 am to 11:59 am), afternoon (12:00 pm to 5:59 pm), or evening (6:00 pm to 10:00 pm)].

### Data analyses

Multilevel analyses were conducted using SUDAAN 10.0. To examine EMA survey compliance patterns (objective #1), multilevel logistic regression analyses tested whether the likelihood of survey non-response (unanswered versus answered) varied as a function of day of the week, time of day, sex, age, race/ethnicity, annual household income, and weight status. To examine accelerometer non-wear patterns, multilevel logistic regression analyses tested whether the likelihood of accelerometer non-wear (versus wear) during the matched EMA prompt varied as a function of day of the week, time of day, sex, age, race/ethnicity, annual household income, and weight status. Multilevel linear regression investigated whether EMA survey non-response was related to concurrent MVPA and SA (measured by accelerometer; objective #2). Multilevel repeated measures models conducted in SAS PROC MIXED compared MVPA and SA (in minutes measured by accelerometer) during the 15-min interval before and 15 min interval after each EMA survey response, stratified by EMA-reported (1) physical activity, and (2) sedentary behavior, respectively (objective #3). The construct validity of EMA survey reports of physical activity and sedentary behavior (objective #4) was tested through multilevel linear regression analyses with the EMA-reported main activity type (nine-level categorical variable) as the independent variable and concurrent MVPA and SA (measured by accelerometer) as the dependent variables. For the model testing differences in SA, focused pairwise contrasts were examined between the three EMA-reported sedentary behaviors (reading/computer, watching TV/movies, sitting) and two slightly higher intensity EMA-reported activities (cooking/chores, childcare/helping with children).

All models were initially stratified by weight status (underweight/normal weight versus overweight/obese). When no group differences were found, results are presented for the entire sample with the groups combined and weight status included as covariate. All models also controlled for sex, age, annual household income, and race/ethnicity (Hispanic versus non-Hispanic; at level two). Adjusted Wald *F* statistics and associated *P* values were used to determine the statistical significance of each factor in the regression analyses. Predicted marginal means (i.e., adjusting for all of the other model covariates) were generated from the linear regressions (Korn and Graubard, 1999).

## Results

### Data availability

**Figure 3:**
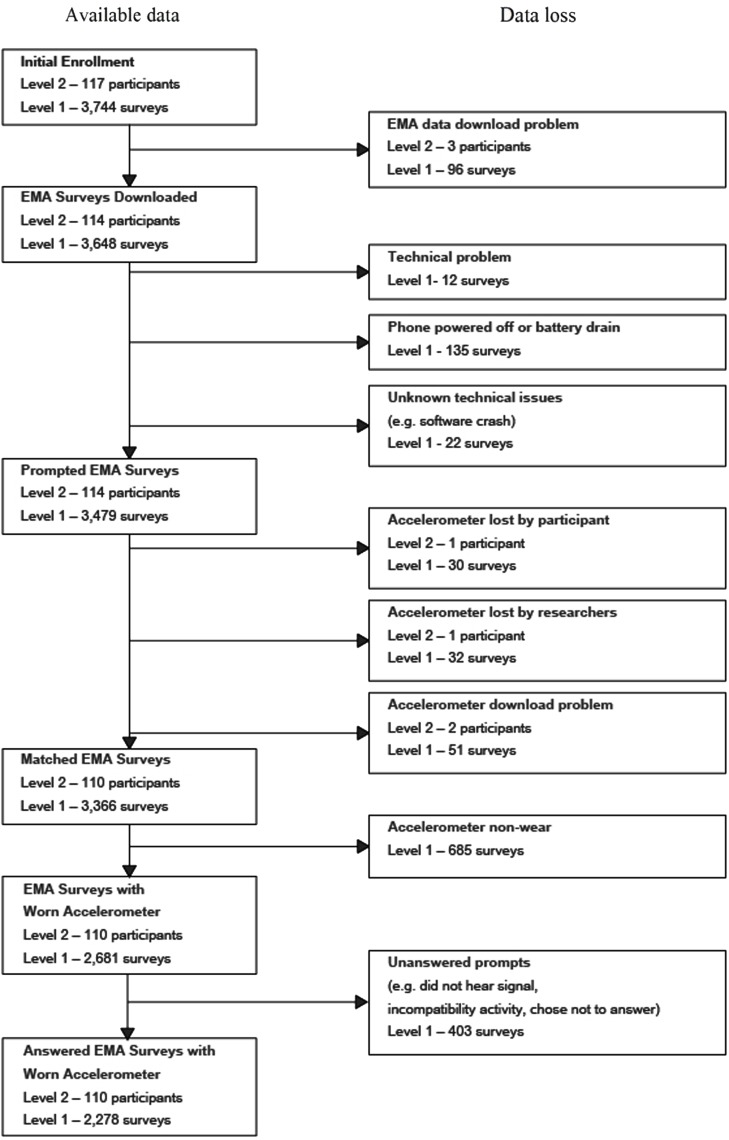
**Flow chart of data availability**. Level 1 represents the number of electronic Ecological Momentary Assessment (EMA) surveys, and Level 2 represents the number of participants. EMA Surveys Downloaded = the number of EMA surveys successfully downloaded from the mobile phone. Prompted EMA Surveys = the number of EMA surveys with a time and date record of being prompted. Matched EMA Surveys = the number of prompted EMA surveys that could be time-matched to available accelerometer data. EMA Surveys with Worn Accelerometer = the number of prompted EMA surveys that could be time-matched to data indicating accelerometer wear during that period. Accelerometer wear was defined as greater than zero activity counts in the ±15 min window of each electronic EMA survey prompt. Answered EMA Surveys with Worn Accelerometer = the number of answered EMA surveys that could be time-matched to data indicating accelerometer wear during that period.

A flow chart displaying data availability and sources of missing data is shown in Figure 3. No mobile phones were lost, stolen, or damaged. EMA data for three participants was mistakenly deleted prior to being downloaded. Eleven participants had insufficient data (<50% available) due to major technical problems with the phone (*n* = 6), a lack of understanding of the mobile phone instructions (*n* = 2), a job where responding to the surveys was not tolerated (*n* = 1), an unusually busy schedule during the weekend monitored (*n* = 1), and disinterest (*n* = 1). Eight of these individuals agreed to participate in a retrial. For these individuals, data from the retrial (not the original trial) are shown in Figure 3 and used in the analyses. The six participants with insufficient data due to a major technical problem with the phone did not differ from the rest of the sample in terms of any demographic factors or weight status.

Of the 3,648 programmed EMA surveys (32 surveys each across 114 participants), 135 (3.7%) EMA surveys were not prompted due to the phone being powered off or battery drain, and 22 (0.6%) were not prompted due to unknown technical problems. Two accelerometers were lost prior to data download (*n* = 1 lost by a participant and *n* = 1 lost by the researchers). Accelerometer data were unavailable for two participants due to problems downloading the devices. Also, accelerometers were not worn (as determined by 0 total activity counts during the ±15 min of the EMA survey prompts) during 685 of the EMA survey prompts. The likelihood of accelerometer non-wear did not vary as a function of day of the week, sex, age, race/ethnicity, and annual household income. However, accelerometer non-wear was more likely to occur during the morning (6:30 am to 12:00 pm; 37%) than afternoon (12:00 pm to 6:00 pm; 8%) or evening (6:00 pm to 10:00 pm; 16%; Adj. Wald *F* = 41.17, df = 2, *p* < 0.001). Accelerometer non-wear was also higher among obese participants (27%) than underweight/normal weight (17%) and overweight (19%) participants (Adj. Wald *F* = 4.29, df = 2, *p* = 0.016). Of the 2,681 EMA survey prompts that were matched with data from a worn accelerometer, a total of 403 prompts were unanswered, resulting in an analytic sample size of 2,278 answered EMA surveys across 110 participants.

### Descriptive statistics

Demographic characteristics for the analytic sample (*N* = 110) are shown in Table 1. Participants were mainly female (72.5%), married (66.1%), and overweight/obese (61.82%). Individuals ranged in age from 27 to 73 years with an average age of 40.4 years (SD = 9.74). The sample was racially/ethnically diverse with 30.3% Hispanic/Latino. Twenty-four percent had an annual household income less than $40,000. Of the EMA surveys answered while wearing an accelerometer, physical activity was reported to be the main activity in 8.6% of EMA surveys, which comprised of Walking (4.4%), Running/Jogging (0.5%), Weight lifting/Strength training (0.4%), Using Cardiovascular Equipment (0.4%), Bicycling (0.3%), Other (2.6%). Sedentary behavior was reported as the main activity in 39.6% of EMA surveys, which was comprised of Reading/Computer (17.0%), Watching TV/Movies (14.3%), and Something else-Sitting (8.3%). Other main activity types indicated were as follows: Eating/Drinking (12.5%), Talking on the phone (6.7%), Cooking/Chores (8.0%), Riding in a car (11.1%), Childcare/Helping children (4.9%), and Something else-Standing (5.0%), and Something else-Walking (3.3%).

**Table 1:** **Participant characteristics**.

	*n* (%)
**SEX^a^**	
Male	30 (27.5)
Female	79 (72.5)
**MARITAL STATUS^b^**	
Never married	21 (19.3)
Married	72 (66.1)
Separated/divorced/widowed	16 (14.7)
**ANNUAL HOUSEHOLD INCOME^c^**	
Less $40,000	25 (23.6)
$40,000–$70,000	24 (22.6)
$70,001–$90,000	27 (25.5)
Above $90,000	30 (28.3)
**RACE^d^**	
African–American	8 (7.8)
Asian	28 (27.2)
White/Caucasian	48 (46.6)
Biracial/Mixed	9 (8.7)
Other	10 (9.7)
**ETHNICITY**	
Hispanic/Latino	33 (30.3)
Non-Hispanic/Latino	77 (69.7)
**WEIGHT STATUS**	
Underweight/Normal Weight	42 (38.2)
Overweight	34 (30.9)
Obese	34 (30.9)

*^a^Sex data missing for one participant; ^b^Marital status data missing for one participant; ^c^Annual household income missing for four participants; ^d^Race data missing for seven participants*.

### Unanswered EMA prompts

On average, participants answered 82% (range 25–100%) of EMA prompts. The average EMA survey answer rate for prompts while wearing an accelerometer was 85%. The likelihood of EMA survey non-response did not vary as a function of day of the week, time of day, sex, age, race/ethnicity, annual household income, or weight status. When examining the ±15 min of each EMA survey prompt, SA did not differ between answered (pred. marginal mean = 19.46, SE = 0.25 min) and unanswered (pred. marginal mean = 20.02, SE = 0.25 min) prompts. For MVPA, the results differed by weight status group. Unanswered EMA prompts had greater MVPA (± 15 min; pred. marginal mean = 1.35, SE = 0.34 min) than answered EMA prompts (pred. marginal mean = 0.60, SE = 0.11 min; Adj. Wald *F* = 4.91, df = 1, *p* = 0.029) for underweight and normal weight participants, indicating that activity level might influence likelihood of responding. However, for overweight/obese participants, MVPA did not differ between answered (pred. marginal mean = 0.91, SE = 0.11 min) as compared with unanswered (pred. marginal mean = 0.85, SE = 0.16 min) EMA survey prompts.

### Extent to which EMA surveys disrupted physical activity and sedentary behavior

To determine whether the act of answering an EMA survey disrupted a participant’s ongoing activity, we compared MVPA and SA (measured by accelerometer) in the 15-min before versus the 15-min after each answered EMA survey. When physical activity was the main activity reported by EMA, results showed MVPA minutes did not differ during the 15-min interval before (*M* = 1.38, SD = 3.20 min) as compared to the 15-min interval after (*M* = 1.28, SD = 2.98 min) the answered survey prompt. For sedentary behavior, the results differed by weight status. Overweight and obese participants engaged in less SA during the 15-min before the prompt (*M* = 11.04, SD = 3.34 min) as compared with the 15-min after the prompt (*M* = 11.44, SD = 3.11 min; β = −0.3579, *p* = 0.035) when sedentary behavior was the main activity reported by EMA. For underweight and normal weight individuals, SA did not differ during the 15-min interval before (*M* = 11.37, SD = 3.09 min) as compared to the 15-min interval after (*M* = 11.68, SD = 2.95 min) the answered EMA survey prompt reporting sedentary behavior as the main activity.

### Validity of EMA activity responses

**Figure 4:**
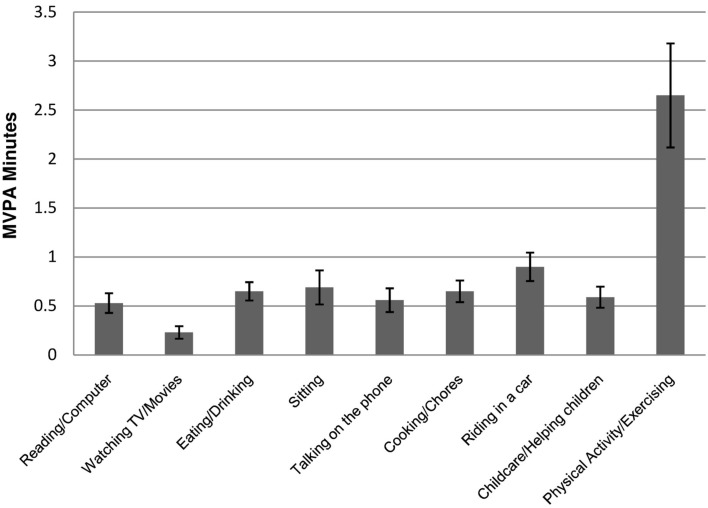
**Mean moderate-to-vigorous physical activity (MVPA) minutes by activity categories self-reported through ecological momentary assessment (EMA)**. MVPA was recorded by accelerometer in the ±15 min window of each answered EMA survey prompt. Values represent the predicted marginal means generated through multilevel linear regressions, which adjusted for sex, age, race/ethnicity, annual household income, and weight status. Standard error (SE) bars are shown. Non-overlapping SE bars indicate a statistically significant difference between means at *p* < 0.05.

**Figure 5:**
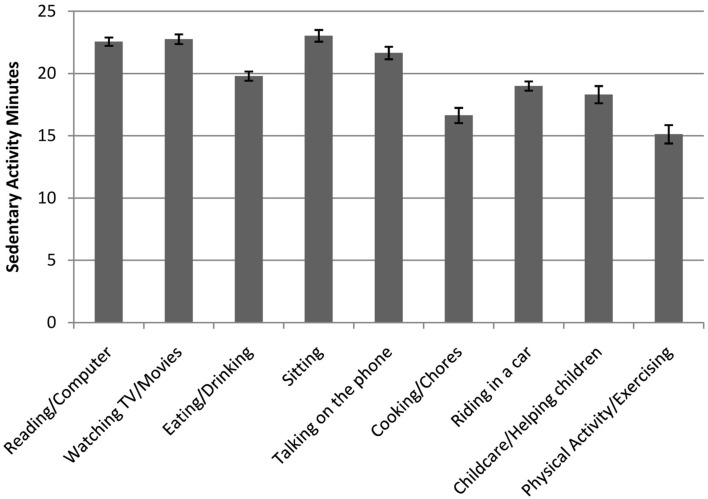
**Mean sedentary activity (SA) minutes by activity categories self-reported through ecological momentary assessment (EMA)**. SA was recorded by accelerometer in the ±15 min window of each answered EMA survey prompt. Values represent the predicted marginal means generated through multilevel linear regressions, which adjusted for sex, age, race/ethnicity, annual household income, and weight status. Standard error (SE) bars are shown. Non-overlapping SE bars indicate a statistically significant difference between means at *p* < 0.05.

The construct validity of EMA activity responses was tested by examining differences in the time-matched MVPA and SA (measured by accelerometer in the ±15 min) by EMA-reported main activity categories. MVPA significantly differed across EMA-reported activities (Adj. Wald *F* = 5.63, df = 8, *p* < 0.001). MVPA was higher for EMA surveys reporting physical activity than any other type of activity (*p*s < 0.001; see Figure 4). SA also differed across EMA-reported activities (Adj. Wald *F* = 28.75, df = 8, *p* < 0.001). SA was higher for EMA surveys reporting computer/reading versus cooking/chores (Adj. Wald *F* = 83.37, df = 1, *p* < 0.001), computer/reading versus childcare/helping with children (Adj. Wald *F* = 38.26, df = 1, *p* < 0.001), watching TV/movies versus cooking/chores (Adj. Wald *F* = 87.22, df = 1, *p* < 0.001), watching TV/movies versus childcare/helping with children (Adj. Wald *F* = 35.68, df = 1, *p* < 0.001), sitting versus cooking/chores (Adj. Wald *F* = 98.05, df = 1, *p* < 0.001), and sitting versus childcare/helping with children (Adj. Wald *F* = 36.46, df = 1, *p* < 0.001; see Figure 5).

## Discussion

Ecological Momentary Assessment of physical activity and sedentary behavior using real-time electronic surveys displayed on mobile phones can reduce recall biases, improve ecological validity, and provide information about specific activity types. Results from the current study support the feasibility and acceptability of 4-day signal-contingent EMA protocol consisting of eight randomly prompted electronic surveys per day. Although EMA has the potential to introduce participant burden, participants answered approximately 82% of the electronic EMA surveys that were prompted. No mobile phones were lost, stolen, or damaged. Rates of missing accelerometer data due to device loss, downloading complications, and non-wear were greater than rates of missing EMA data.

Although some EMA data were lost due to mobile phone software and hardware problems, these instances occurred at random, and the data could be recovered through retrial. All participants who experienced a major technical problem resulting in greater than 50% data loss agreed to participate in a retrial, through which sufficient data was obtained. It is expected that data loss through major technical problems will be reduced in the future as the memory and processing capabilities on mobile phones will be improved through enhanced technology. Data loss due to the phone being powered off, on the other hand, may not occur at random. Future research should seek to identify strategies to collect workday EMA data from these individuals through auto-initiated surveys with short-term recall during work breaks and immediately after work shifts are completed. Data loss due to the phone being powered off may also be more common among individuals with unusual sleep and wake schedules. Future studies may remedy this problem by tailoring the EMA prompting period to individual sleep cycles.

Ecological momentary assessment survey response rates were consistent across sociodemographic and economic groups and comparable to other EMA studies (Hufford et al., 2002; Rofey et al., 2010). However, there was some evidence suggesting that underweight and normal weight individuals (40% of the sample) were less inclined to answer EMA survey prompts while engaging in higher levels of physical activity. From these data, it is unclear whether underweight and normal weight individuals were less likely to carry the phone with them while exercising or carried the phone while exercising but were less likely to answer the prompted surveys at those times. Interestingly, concurrent activity levels did not differ between answered and unanswered surveys for overweight and obese individuals (60% of the sample) when reporting physical activity. It is possible that underweight and normal weight individuals are more likely to engage in types of physical activities (e.g., road bicycling, team sports) where responding to an EMA survey would be difficult. Overweight and obese individuals perform more physical activity through brisk walking (Spees et al., 2012), which may be more compatible to EMA survey response. To promote EMA of higher intensity activities, a context-sensitive approach may be taken (Intille et al., 2012), which utilizes information from built-in or external motion sensors, to automatically prompt EMA surveys during natural activity breaks or immediately after the activity concludes.

Results indicated that the act of responding to the EMA survey did not disrupt physical activity. MVPA levels did not differ before and after the moment of the completed EMA survey, suggesting that individuals resumed their prior level of physical activity. However, overweight and obese individuals performed higher levels of SA after completing EMA surveys where they reported a sedentary behavior as their main activity. It is possible that the mere act of asking overweight and obese participants to self-report their current level of activity may compel them to persist at that behavior as has been suggested in previous work (Godin et al., 2008).

Another objective of this study was to test the criterion validity of participants’ real-time self-reports of activity type. Results indicated that time-matched objective activity data (measured by accelerometer) corresponded with EMA self-reports of current activity levels. MVPA and SA in the ±15 min window surrounding each EMA prompt were greater for surveys reporting physical activity and sedentary behavior, respectively, than those reporting other types of behavior. These findings alleviate concerns that participants regularly provide untruthful, socially desirable responses; report activities that they have recently performed (instead of their current activity); or answer surveys haphazardly out of haste. Overall, these results provide evidence for the validity of real-time data capture techniques to measure physical activity and sedentary behaviors through self-report.

This study had a few limitations. First, the EMA protocol only monitored behavior on two weekend days and two weekdays (Monday and Tuesday). It is possible that the weekend selected or those particular weekdays are not representative of participants’ usual daily lives. A longer monitoring period would be preferred but might introduce greater participant burden. Second, activity thresholds for sedentary behavior are not consistently defined in the literature. Different sedentary activity thresholds may yield different results. Third, the current main activity item did not distinguish between computer use performed for productive (i.e., related to paid work or household management) and leisure (e.g., social networking, Internet surfing, videos) purposes, which may be differentially targeted for intervention. Fourth, these EMA data do not indicate duration of activities. Also, not all physical activity and sedentary behavior was captured due to the random signal-contingent sampling procedure. The overall purpose of the EMA protocol was not to provide a measure of total physical activity and sedentary behavior during the monitoring period. Instead, the goal was to sample the occurrence of specific behaviors that can be linked to other time-intensive EMA measures such as mood and context. Lastly, the results may not be applicable to highly physically active individuals or those from high income households, or non-English speakers as they were excluded from the study.

Findings generally support the acceptability and validity of a 4-day signal-contingent EMA protocol using mobile phones to measure physical activity and sedentary behavior in adults. However, some MVPA may be missed among underweight and normal weight individuals.

## Conflict of Interest Statement

The authors declare that the research was conducted in the absence of any commercial or financial relationships that could be construed as a potential conflict of interest.

## Footnotes

^1^http://www.itu.int/ITU d/ict/publications/idi/2010/Material/MIS_2010_Summary_E.pdf

^2^http://myexperience.sourceforge.net/

## References

[B1] Belcher B. R. Berrigan D. Dodd K. W. Emken B. A. Chou C. P. Spruijt-Metz D. (2010). Physical activity in US youth: effect of race/ethnicity, age, gender, and weight status. Med. Sci. Sports Exerc. 42, 2211–2221 10.1249/MSS.0b013e3181e1fba9 21084930 PMC3242154

[B2] Colley R. Gorber S. C. Tremblay M. S. (2010). Quality control and data reduction procedures for accelerometry-derived measures of physical activity. Health Rep. 21, 63–69 20426228

[B3] Conroy D. E. Elavsky S. Hyde A. L. Doerksen S. E. (2011). The dynamic nature of physical activity intentions: a within-person perspective on intention-behavior coupling. J. Sport Exerc. Psychol. 33, 807–827 22262706 10.1123/jsep.33.6.807 PMC4137572

[B4] Dunton G. F. Atienza A. A. (2009). The need for time-intensive information in healthful eating and physical activity research: a timely topic. J. Am. Diet. Assoc. 109, 30–35 10.1016/j.jada.2009.06.084 19103320

[B5] Dunton G. F. Atienza A. A. Castro C. M. King A. C. (2009). Using ecological momentary assessment to examine antecedents and correlates of physical activity bouts in adults age 50+ years: a pilot study. Ann. Behav. Med. 38, 249–255 10.1007/s12160-009-9141-4 20052568 PMC7155921

[B6] Dunton G. F. Liao Y. Intille S. S. Spruijt-Metz D. Pentz M. (2011). Investigating children’s physical activity and sedentary behavior using ecological momentary assessment with mobile phones. Obesity (Silver Spring) 19, 1205–1212 10.1038/oby.2010.302 21164502

[B7] Froehlich J. Chen M. Y. Consolvo S. Harrison B. Landay J. A. (2007). “Myexperience: a system for in situ tracing and capturing of user feedback on mobile phones,” in Proceedings of the 5th International Conference on Mobile Systems, Applications and Services, San Juan, 57–70

[B8] Godin G. Sheeran P. Conner M. Germain M. (2008). Asking questions changes behavior: mere measurement effects on frequency of blood donation. Health Psychol. 27, 179–184 10.1037/0278-6133.27.2.179 18377136

[B9] Haskell W. L. (2012). Physical activity by self-report: a brief history and future issues. J. Phys. Act. Health 9(Suppl. 1), S5–S10 22287448 10.1123/jpah.9.s1.s5

[B10] Hawkins M. S. Storti K. L. Richardson C. R. King W. C. Strath S. J. Holleman R. G. Kriskariska A. M. (2009). Objectively measured physical activity of USA adults by sex, age, and racial/ethnic groups: a cross-sectional study. Int. J. Behav. Nutr. Phys. Act. 6, 31 10.1186/1479-5868-6-31 19493347 PMC2701914

[B11] Healy G. N. Dunstan D. W. Salmon J. Cerin E. Shaw J. E. Zimmet P. Z. Owen N. (2008). Breaks in sedentary time: beneficial associations with metabolic risk. Diabetes Care 31, 661–666 10.2337/dc07-2046 18252901

[B12] Hufford M. R. Shields A. L. Shiffman S. Paty J. Balabanis M. (2002). Reactivity to ecological momentary assessment: an example using undergraduate problem drinkers. Psychol. Addict. Behav. S16, 205–211 10.1037/0893-164X.16.3.205 12236455

[B13] Intille S. S. Lester J. Sallis J. F. Duncan G. (2012). New horizons in sensor development. Med. Sci. Sports Exerc. 44(Suppl. 1), S24–S31 10.1249/MSS.0b013e3182399c7d 22157771 PMC3245518

[B14] Kanning M. Schlicht W. (2010). Be active and become happy: an ecological momentary assessment of physical activity and mood. J. Sport Exerc. Psychol. 32, 253–261 20479481 10.1123/jsep.32.2.253

[B15] Kaplan W. A. (2006). Can the ubiquitous power of mobile phones be used to improve health outcomes in developing countries? Global Health 2, 9 16719925 10.1186/1744-8603-2-9 PMC1524730

[B16] Korn E. L. Graubard B. I. (1999). Analysis of Health Surveys. New York: Wiley

[B17] Kosaraju A. Barrigan C. R. Poropatich R. K. Casscells S. W. (2010). Use of mobile phones as a tool for United States health diplomacy abroad. Telemed. J. E. Health 16, 218–222 10.1089/tmj.2009.0095 20156128

[B18] Loney T. Standage M. Thompson D. Sebire S. J. Cumming S. (2011). Self-report vs. objectively assessed physical activity: which is right for public health? J. Phys. Act. Health 8, 62–70 21297186 10.1123/jpah.8.1.62

[B19] Merom D. Bowles H. Bauman A. (2009). Measuring walking for physical activity surveillance – the effect of prompts and respondents’ interpretation of walking in a leisure-time survey. J. Phys. Act. Health 6(Suppl. 1), S81–S88 19998853 10.1123/jpah.6.s1.s81

[B20] Nicaise V. Marshall S. Ainsworth B. E. (2011). Domain-specific physical activity and self-report bias among low-income Latinas living in San Diego County. J. Phys. Act. Health 8, 881–890, 21885878 10.1123/jpah.8.7.881

[B21] Patrick K. Griswold W. G. Raab F. Intille S. S. (2008). Health and the mobile phone. Am. J. Prev. Med. 35, 177–181 10.1016/j.amepre.2008.05.001 18550322 PMC2527290

[B22] Physical Activity Guidelines Advisory Committee (2008). Physical Activity Guidelines Advisory Committee Report Washington, DC: U.S. Department of Health and Human Services

[B23] Rofey D. L. Hull E. E. Phillips J. Vogt K. Silk J. S. Dahl R. E. (2010). Utilizing ecological momentary assessment in pediatric obesity to quantify behavior, emotion, and sleep. Obesity (Silver Spring) 18, 1270–1272 10.1038/oby.2009.483 20019675 PMC2896245

[B24] Spees C. K. Scott J. M. Taylor C. A. (2012). Differences in amounts and types of physical activity by obesity status in US adults. Am. J. Health Behav. 36, 56–65 10.5993/AJHB.36.1.6 22251783 PMC3888549

[B25] Thomas J. G. Bond D. S. Ryder B. A. Leahey T. M. Vithiananthan S. Roye G. D. Wing R. R. (2011). Ecological momentary assessment of recommended postoperative eating and activity behaviors. Surg. Obes. Relat. Dis. 7, 206–212 10.1016/j.soard.2010.10.007 21130703

[B26] Troiano R. P. Berrigan D. Dodd K. W. Masse L. C. Tilert T. McDowell M. (2008). Physical activity in the United States measured by accelerometer. Med. Sci. Sports Exerc. 40, 181–188 10.1249/01.mss.0000322246.48549.fc 18091006

[B27] Troiano R. P. Pettee Gabriele K. K. Welk G. J. Owen N. Sternfeld B. (2012). Reported physical activity and sedentary behavior: why do you ask? J. Phys. Act. Health 9(Suppl. 1), S68–S75 22287450 10.1123/jpah.9.s1.s68

[B28] Tucker J. M. Welk G. J. Beyler N. K. (2011). Physical activity in U.S.: adults compliance with the physical activity guidelines for Americans. Am. J. Prev. Med. 40, 454–461 10.1016/j.amepre.2010.12.016 21406280

